# Association between C-reactive protein level and subsequent risk of ovarian cancer: A meta-analysis of 13 cohorts in 1,852 ovarian cancer patients

**DOI:** 10.1097/MD.0000000000018821

**Published:** 2020-01-31

**Authors:** Yan Wang, Zhiming Zhang, Jing Wang, Xiaowei Zhang

**Affiliations:** a Department of Gynecology; b Department of Clinical Laboratory, Xi’an Central Hospital; c Department of Oncology of Gynecology, Shaanxi Provincial Cancer Hospital; d Department of oncology, Xi’an Central Hospital, China.

**Keywords:** c-reactive protein, disease risk, meta-analysis, ovarian cancer

## Abstract

**Background:**

Though studies have shown association between C-reactive protein (CRP) level and the risk of ovarian cancer (OC), there have been some inconsistencies. The current metaanalysis was conducted to study the relationship between CRP and OC.

**Patients and methods:**

Three electronic databases of PubMed, Embase, and Cochrane Library were searched for prospective studies of OC from inception till May 2018. Relative risk (RR) was summarized using random-effects model, and the results of sensitivity, subgroup analyses, and publication biases were also calculated.

**Results:**

A total of 13 cohorts involving 1,852 OC patients were included for the final meta-analysis. The summary RRs indicated that high CRP was associated with an increased risk of all invasive OC (RR:1.36; 95% confidence interval [CI]:1.03–1.80; *P* = .032), while moderate CRP showed no significant impact on the risk of all invasive OC compared with low CRP (RR:1.17; 95% CI:0.97–1.41; *P* = .107). High (RR: 1.42; 95% CI: 0.85–2.37; *P* = .183) or moderate (RR: 1.29; 95% CI: 0.94–1.77; *P* = .119) CRP levels showed little or no effect on serous OC. Similarly, no significant differences for the comparisons of high versus low (RR: 1.82; 95% CI: 0.27–12.42; *P* = .540) or moderate versus low (RR: 0.72; 95% CI: 0.31–1.69; *P* = .455) CRP levels for the risk of mucinous OC were observed. Moreover, high (RR: 0.58; 95% CI: 0.13–2.54; *P* = .471) or moderate (RR: 0.81; 95% CI: 0.44–1.47; *P* = .484) CRP levels were not associated with the risk of endometrioid OC compared with low CRP levels.

**Conclusion:**

High CRP levels were associated with increased risk of invasive OC. The risk of other OC types with CRP levels showed no association.

## Abstract 2

Supplemental Digital Content is available in the text

## 1 Introduction

Ovarian cancer (OC) is 1 of the leading causes of gynecologic cancer death in women worldwide, accounting for 295,414 women and causing greater than 184,799 annual deaths.^[1,2]^ Although surgical cytoreduction and chemotherapy are established treatment strategies for patients with OC, the mean age-standardized 5-year survival rate is just about 45% either due to treatment resistance or late diagnosis.^[3]^

OC has poor prognosis; multi-modal screening using carbohydrate antigen (CA)125 values and transvaginal ultrasound have been widely used for predicting OC risk.^[4]^ Several studies have illustrated the role of inflammation in promoting ovarian tumorigenesis and cancer progression, and the involvement of pro-inflammatory cytokines in the pathogenesis of OC.^[5–9]^ C-reactive protein (CRP), an acute-phase protein, is an indicator of infectious or inflammatory conditions and considered as a prognostic factor in different types of cancer.^[10,11]^

Many studies have established an association of CRP with OC. While normal CRP levels are below 3.0 mg/L, the levels in OC patients can rise up to 14.32 mg/L.^[12]^ A meta-analysis suggests that increased CRP levels rather than circulating proinflammatory cytokines might contribute to the etiology of OC.^[13]^ Heterogeneity in terms of CRP levels and of different types of tumors have been observed. Women with CRP concentrations >10 mg/L have a 67% risk of OC especially mucinous and endometrioid carcinoma, and likely for serous and clear cell carcinoma since statistical significance was not observed.^[14]^ Higher ratios of CRP/Albumin (≥0.68) was related with OC of advanced stage, residual tumor, ascites, higher serum CA-125 level, glasgow prognostic score (GPS), modified GPS and poor overall survival.^[15]^ About 23% of OC patients suffer from chronic inflammation as indicated by elevated CRP concentrations and the risk of developing OC among women in the highest third of the distribution of CRP compared with those in the lowest third was 1.72 (95% confidence interval [CI]: 1.06–2.77).^[16]^ A recent meta-analysis revealed 34% increased risk of OC when comparing women in the top tertile of CRP levels with those in the bottom tertile (1.34 [95% CI: 1.06–1.70] ) and the risk doubled in women with CRP levels >10 mg/L,^[17]^ while another meta-analysis showed that increased levels of CRP, but not circulating IL6, TNFα, or soluble TNFR2, have significant relationship with OC risk.^[13]^

Numerous studies have demonstrated a relationship between OC risk and CRP levels with various OR values ranging from 1.09 to 2.33 for the highest and lowest tertile.^[16,18–21]^ While a previous study shows negative correlation, it has been reported that increased CRP levels is still a risk factor and that chronic inflammation plays a part in OC.^[22]^ However, not many studies have explored if CRP levels could affect a specific type of OC. Further, the range of serum CRP level and the cutoff values for the categories differed among the studies. We therefore attempted to comprehensively examine the available prospective observational studies to measure the association between serum CRP level and OC and whether these relationships differed between studies or patients with specific characteristics were also calculated.

## 2 Methods

### 2.1 Data sources, search strategy, and selection criteria

This study was conducted and reported according to the meta-analysis of observational studies in epidemiology protocol.^[23]^ Studies that investigated the association of CRP with the risk of OC were eligible for inclusion in this study. Searches on electronic databases were performed without any restrictions on language and publication status. PubMed, Embase, and Cochrane Library were searched for studies published from inception to May 2018. The core keywords used for searching the studies were (“Creactive protein” or “C-reactive protein” or “CRP”) and (“ovarian cancer” or “ovarian carcinoma”). Potentially eligible studies were searched from the reference lists of the papers included in the present study. This study was a meta-analysis so ethical approval was waived or not necessary, and informed consent can’t be obtained.

Studies were included if they met the following inclusion criteria:

(1) Study with a prospective observational design (prospective cohort or nest prospective case-control study);
(2) Study investigated the association of serum CRP level and the risk of OC;
(3) Study reporting the effect estimates (risk ratio [RR], hazard ratio [HR], or odds ratio [OR] and 95% CIs) for comparison of various categories of serum CRP levels.

Studies with retrospective design (traditional case-control or retrospective cohort design) were excluded as various confounding factors could bias the results. The literature search and study selection processes were undertaken by 2 authors and any disagreements were resolved by group discussion until a consensus was reached.

### 2.2 Data collection and quality assessment

Data extraction and quality assessments were conducted independently by 2 authors, and any inconsistencies between them were examined and adjudicated independently by third author referring to the original studies. The data items collected included the first author's surname, study group's name, publication year, country, study design, study year, assessment of exposure, number of OC cases, age, cutoff values, reported outcomes, and adjusted factors. Effect estimates were selected by maximally adjusting the potential confounders in case a study reported several multivariable adjusted effect estimates. The newcastle-ottawa scale (NOS) was used to assess the methodological quality, which was based on selection (4 items), comparability (1 item), and outcome (3 items), and a “star system” (range, 0–9) has been developed for assessment.^[24]^

### 2.3 Statistical analysis

The relationship between serum CRP level and the risk of OC was based on the effect estimates and corresponding 95%CI in each individual study. The summary RRs and 95%CIs for the high (>3.0 mg/L) or moderate (between low and 3.0 mg/L) versus low serum CRP levels were calculated using the random-effects model.^[25,26]^ The value assigned to each serum CRP level category was the mid-point for closed categories and median for open categories (assuming a normal distribution for serum CRP level). Heterogeneity among the included studies was calculated using *I*-square and *Q* statistic, and *P* < .10 was regarded as significant heterogeneity.^[27,28]^ Sensitivity analyses for all invasive OC and serous OC were conducted to evaluate the impact of single cohort in the overall analysis.^[29]^ Subgroup analyses were conducted for all invasive OC based on publication year, study design, adjusted body mass index (BMI), and adjusted contraceptive use. Publication biases for investigated outcomes were calculated using funnel plots, Egger, and Begg tests,^[30,31]^ and if significant publication bias was observed, then a trim and fill test was conducted to adjust the pooled results.^[32]^*P*-values for overall, sensitivity, and subgroup analyses are 2-sided, and *P*-values < .05 were regarded as statistically significant. All statistical analyses were performed using STATA software (version 10.0; Stata Corporation, College Station, TX).

## 3 Results

### 3.1 Literature search

**Figure 1:**
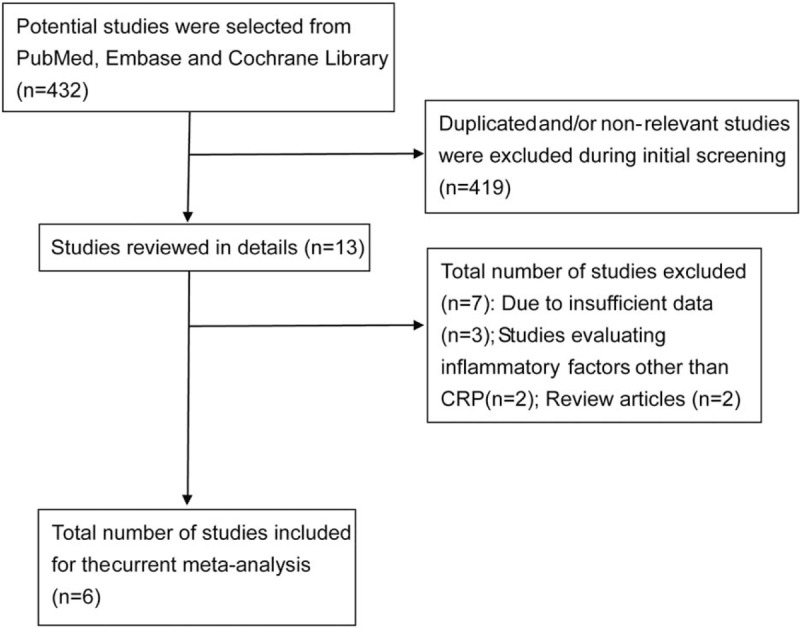
Flow-chart showing details of the study-selection process.

The results of the study-selection process are shown in Figure 1. Four hundred thirty-two articles were identified from the initial electronic search. Of these, 419 were excluded due to non-relevance to the current study and/or duplication. A total of 13 studies showed detailed evaluations, where 7 were excluded due to insufficient data (n = 3), evaluated inflammation factors other than CRP (n = 2), and review articles (n = 2). Finally, 6 studies involving 13 cohorts were included in the final analysis. ^[16,18–22]^ No additional studies were identified by manual search of the reference lists of these studies. Table 1 summarized the general characteristics of the included studies.

**Table 1:**
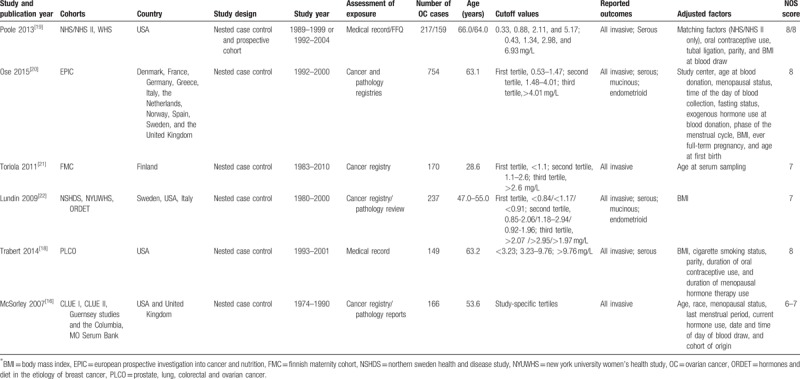
Baseline characteristic of included studies in the systematic review and meta-analysis.

### 3.2 Study characteristics

Six studies involving 13 cohorts with 1852 OC patients were included. Of these, 12 cohorts had nested case-control design, and one cohort had a prospective cohort design. The study period ranged from 1974–2010, and number of OC cases ranged from 149–754 in each study. All these studies were conducted in USA and European countries. Six studies described the risk of invasive OC, 4 studies described serous OC, 2 studies discussed mucinous OC, and 2 studies were dedicated to endometrioid OC. Study quality of the included studies was evaluated by NOS. Nearly all the included cohorts (11/13) scored 7 or 8 and were presented in Table 1.

### 3.3 All invasive OCs

**Figure 2:**
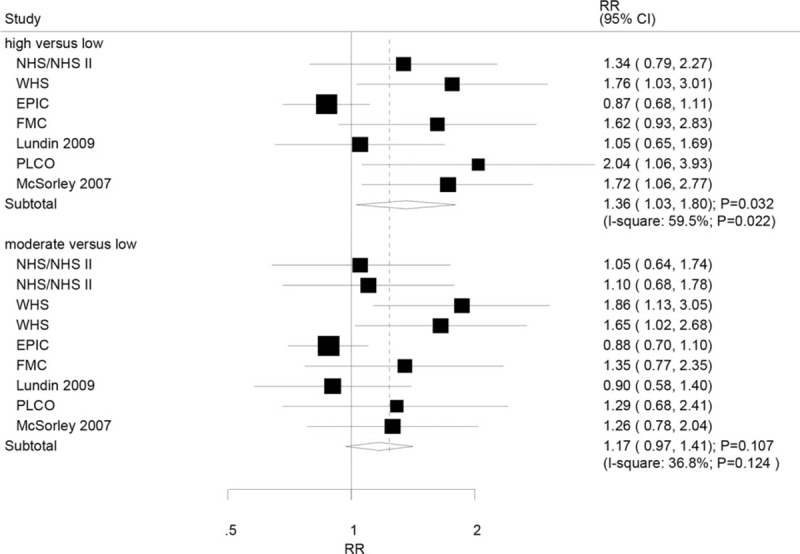
Association between serum CRP levels and the risk of all invasive ovarian cancers. CRP = C-reactive protein.

A total of 6 studies reported an association between high as well as moderate serum CRP level and all invasive OC. The summary RR showed that a high serum CRP level was associated with an increased risk of all invasive OC as compared with low serum CRP level (RR: 1.36; 95%CI: 1.03–1.80; P = 0.032; Fig. 2), but potential evidence of significant heterogeneity was observed (*P* = .022). As a result, a sensitivity analysis was conducted, and after sequential exclusion of each study from the pooled analysis, the conclusion varied due to smaller number of included studies. Subgroup analysis indicated high serum CRP level with greater risk of all invasive OC if the study included a prospective cohort or the study did not adjust for BMI (Table S1 and S2).

Further, pooled analysis results indicated that there was no association between moderate serum CRP level and all invasive OC (RR: 1.17; 95% CI: 0.97–1.41; *P* = .107; Fig. 2), and moderate heterogeneity was observed (*P* = .124). According to the sensitivity analysis, the study by Ose et al,^[20]^ and concluded that the moderate serum CRP level significantly increased the risk of all invasive OC by 26% compared to low serum CRP level (RR, 1.26; 95% CI, 1.05–1.50; *P* = .011; Table 2). Subgroup analysis indicated that the moderate serum CRP level significantly increased the risk of all invasive OCs if the study had prospective cohort design (Table 2).

**Table 2:**
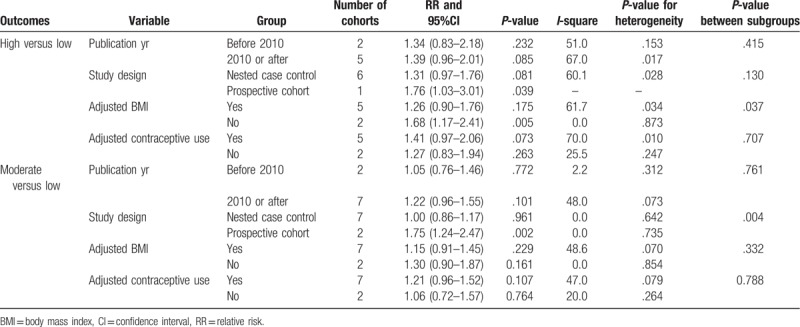
Subgroup analyses for all invasive ovarian cancer.

### 3.4 Serous OC

**Figure 3:**
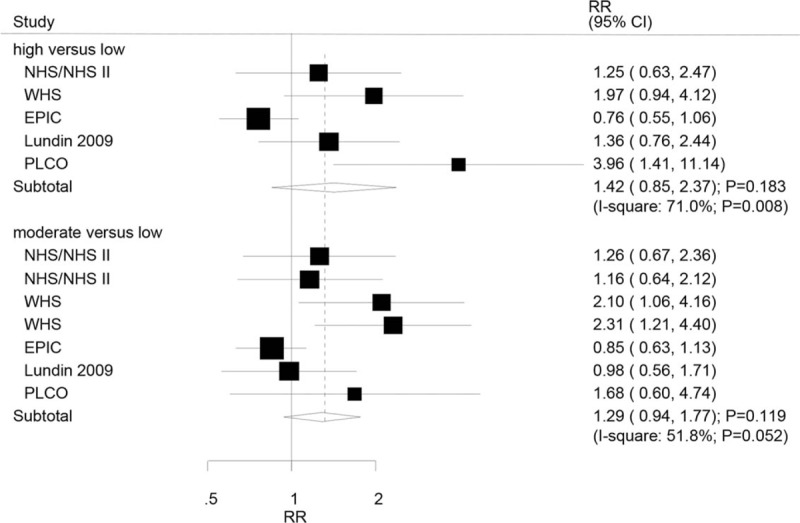
Association between serum CRP levels and the risk of serous ovarian cancer. CRP = C-reactive protein.

A total of 4 studies reported an association between high or moderate serum CRP level and serous OC. There were no significant associations between high (RR: 1.42; 95% CI: 0.85–2.37; *P* = .183) or moderate (RR: 1.29; 95% CI: 0.94–1.77; *P* = .119) serum CRP levels and serous OC (Fig. 3). Significant heterogeneity was observed across the included studies for high or moderate serum CRP levels and the risk of serous OC. Sensitivity analyses indicated that the risk of serous OC increased when the study conducted by Ose et al^[20]^ was excluded (Table S3 and S4).

### 3.5 Mucinous OC

**Figure 4:**
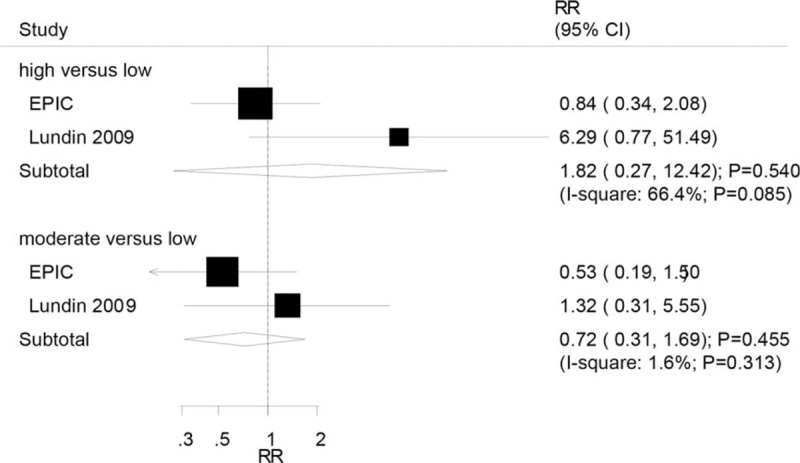
Association between serum CRP levels and the risk of mucinous ovarian cancer. CRP = C-reactive protein.

Two studies reported an association between high or moderate serum CRP levels and mucinous OC. The summary RR indicated that high (RR: 1.82; 95% CI: 0.27–12.42; *P* = .540) or moderate (RR: 0.72; 95% CI: 0.31–1.69; *P* = .455) serum CRP levels were not associated with the risk of mucinous OC (Fig. 4). Significant heterogeneity was observed for high versus low serum CRP levels, while heterogeneity for moderate versus low serum CRP levels was insignificant.

### 3.6 Endometrioid OC

**Figure 5:**
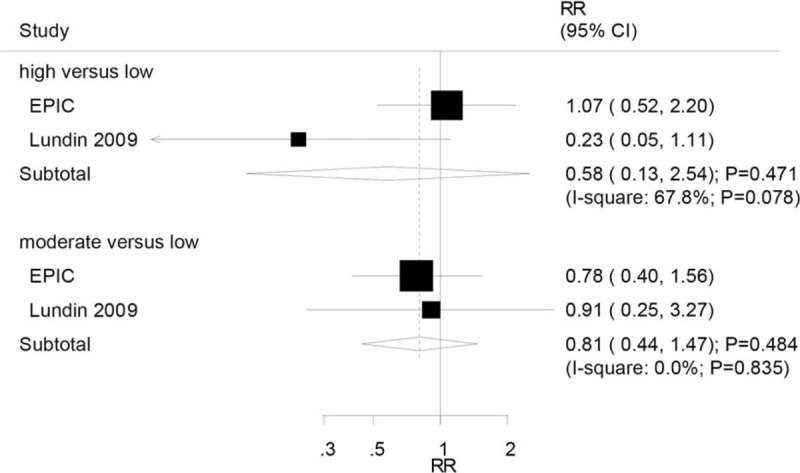
Association between serum CRP levels and the risk of endometrioid ovarian cancer. CRP = C-reactive protein.

Two studies reported an association between high or moderate serum CRP levels and endometrioid OC. It was seen that high serum CRP level was not associated with the risk of endometrioid OC (RR: 0.58; 95% CI: 0.13–2.54; *P* = .471; Fig. 5). Similar results were observed for moderate versus low serum CRP levels and the risk of endometrioid OC (RR: 0.81; 95% CI: 0.44–1.47; *P* = .484; Fig. 5). Substantial heterogeneity was observed across the studies for high versus low serum CRP levels, while no evidence of heterogeneity was detected for moderate versus low serum CRP level.

### 3.7 Publication bias

**Figure 6:**
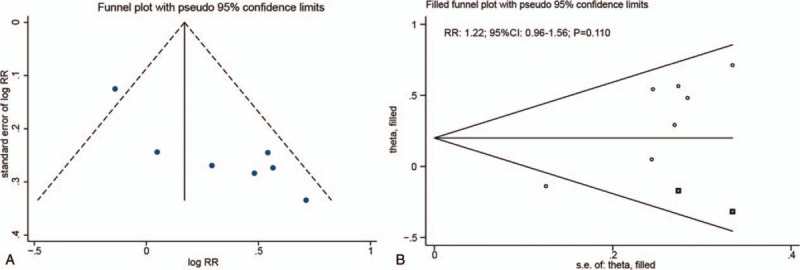
Funnel plot (A) and trim and fill method (B) for high versus low CRP levels and the risk of all invasive ovarian cancers. CRP = C-reactive protein.

**Figure 7:**
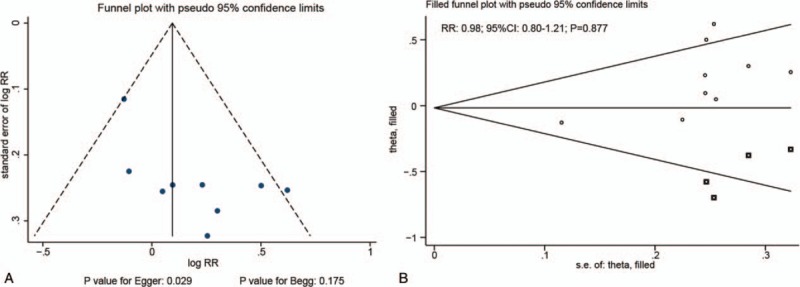
Funnel plot (A) and trim and fill method (B) for moderate versus low CRP levels and the risk of all invasive ovarian cancers. CRP = C-reactive protein.

We noted significant publication biases for high or moderate serum CRP levels and the risk of all invasive OC (Figs. 6 and 7). After using the trim and fill method, we noted that a high (RR: 1.22; 95% CI: 0.96–1.56; *P* = .110) or moderate (RR: 0.98; 95% CI: 0.80–1.21; *P* = .877) serum CRP levels were not associated with the risk of all invasive OC (Figs. 6 and 7)

## 4 Discussion

The current meta-analysis was based on prospective observational studies and, it explored the relationships between serum CRP levels and the outcomes of invasive OC and specific type of OC. This study involved 1852 OC patients from 12 nested case-control studies and 1 prospective cohort study with a broad range of patient characteristics. The results of this study suggested that high versus low serum CRP levels were associated with an increased risk of invasive OC. Further, moderate serum CRP levels have no significant impact on the risk of invasive OC. For specific OC types, there were no significant differences when compared to high or moderate versus low serum CRP levels.

A previous metaanalysis reported that the third tertiles of CRP was associated with an increased risk of OC, while the second tertiles of CRP showed no significant impact on OC risk.^[17]^ Further, increased risk was found in studies dealing with serum CRP, studies conducted in USA, use of high-sensitivity immunotubidimetric assay, use of high-sensitivity CRP, and the duration of the follow-up greater than 10 years. However, our meta-analysis neglected the included studies with various adjusted factors and hence we found high versus low serum CRP levels were associated with an increased risk of invasive OC. Further, the previous study^[17]^ used tertiles of CRP as cutoff values, which might bias the pooled results. Another meta-analysis and found similar results and with same limitations.^[22]^ Both these meta-analyses could not provide the impact of serum CRP levels on the risk of specific type of OC, which we have attempted to explore in our study where we found significant correlation for invasive OC but not for any other type. This also lends credence to the fact that inflammatory responses might be important for the progression of ovarian carcinoma as has been described in various studies.^[8,16,21]^

Another meta-analysis^[14]^ that included 6 cohorts found that OC risk increased by 67% in women with CRP concentrations >10 mg/L increased CRP level, especially for endometrioid and mucinous carcinoma. Since we did not set 10 mg/L as the cutoff value, our results vary with that found in the earlier study. Further, the previous study has accessed data from 6 cohort studies in the OC Cohort Consortium, whereas our method of data collection was different. This might also have given rise to inconsistencies between our study and the previous 1.

The results of our sensitivity analyses suggested that high or moderate serum CRP levels were associated with increased risk of all invasive OC and serous OC after excluding the study conducted by Ose et al^[20]^; this study was performed in the European Prospective Investigation into Cancer and Nutrition cohort that included 23 centers from 10 European countries and reported that CRP level >10 mg/L versus < 1 mg/L was associated with an increased risk of OC. They pointed out the limited role of CRP in ovarian carcinogenesis due to adiposity, a potential risk factor for OC.^[33]^ Similarly, association of higher CRP levels and endometrioid tumors was dependent on BMI.^[34]^ Therefore, serum CRP levels might affect the progression of OC in patients with specific BMI categories. Our subgroup analysis showed high serum CRP level with greater risk of all invasive OC if the study did not adjust for BMI, thereby conforming to the previous studies. Further, a previous study has showed that in serous OC, there was a strong relationship between CRP and interleukin-8.^[19]^ In cancer patients, CRP supposedly expedites angiogenesis based on circulating levels of interleukins and vascular endothelial growth factors.^[15]^ Since we did not consider studies that evaluated inflammatory markers other than CRP, our results show no significant associations between high or moderate serum CRP levels and serous OC.

There were no significant associations of serum CRP levels with the risk of mucinous and endometrioid OC in the present study because they were described in only 2 studies included in our analysis; moreover, we excluded those studies that have described inflammatory factors along with CRP and other lifestyle factors. Further, the event rates of mucinous and endometrioid OC were lower in our study. Therefore, we provided a synthetic review, and these results should be verified in large-scale prospective cohort studies. One study has stated that CRP levels are not a favorable prognosis factor for surgically treated endometrial carcinoma,^[35]^ but proinflammatory cytokines and obesity along with CRP might promote endometrial carcinogenesis.^[36]^ Mucinous OC accounts for 3% to 4% of epithelial OC and a retrospective study that included patients with simple ovarian cyst, benign serous or mucinous cystadenoma reported that serum concentrations of CRP solely or in combination with CA125 may be a useful clinical marker.^[12]^ This differs from our study and could be due to the nature of both the studies and population characteristics.

On another note, preoperative CRP levels were significantly lesser in long-term survivors of OC emphasizing its potential role as prognostic marker for long-term survival.^[37]^ It has also been reported that increased CRP levels contributes to resistance to chemotherapy and poor survival in OC patients.^[38]^ Thus, CRP plays an important role in OC and this could be used to our advantage to detect, treat and predict survival in OC patients.

Our metaanalysis is not without limitations. First, various adjusted factors across the included studies, and the stratified results based on these factors were not available. Second, different cutoff values of serum CRP levels of included studies might affect the summary results since we did not adjust CRP values across studies. Third, the level of CRP was measured using different types of assays in different studies, which might affect the prognosis or detection of OC. Fourth, the analysis was based on published studies, and publication bias among the included studies was statistically significant. Finally, the risk of specific type of OC was obtained from smaller number of studies, and stratified results for these outcomes were not calculated. In spite of the above-mentioned limitations, our meta-analysis provides a temporal overview between the relationship of different types of OC with CRP because prospective studies were used for the current analyses.

In conclusion, the results of this meta-analysis indicated that high serum CRP levels were associated with an increased risk of all invasive OC, while this effect was not observed for moderate versus low serum CRP levels. Further, high or moderate serum CRP levels did not affect the risk of each specific type of OC. These findings could aid to identify women at high risk for OC and appropriate intervention to bring down CRP levels could be made to avoid the progression of OC. Future large-scale prospective studies focusing on specific type of OC and use of uniform category of serum CRP levels should be conducted.

## Author contributions

**Conceptualization:** Yan Wang, Zhiming Zhang, Jing Wang.

**Data curation:** Yan Wang, Zhiming Zhang, Xiaowei Zhang.

**Formal analysis:** Yan Wang, Zhiming Zhang, Xiaowei Zhang.

**Investigation:** Xiaowei Zhang.

**Methodology:** Zhiming Zhang.

**Project administration:** Yan Wang, Zhiming Zhang.

**Software:** Yan Wang, Jing Wang, Xiaowei Zhang.

**Writing – original draft:** Yan Wang, Zhiming Zhang, Jing Wang, Xiaowei Zhang.

**Writing – review and editing:** Yan Wang, Zhiming Zhang, Xiaowei Zhang.

## Supplementary Material

Supplemental Digital Content

## Supplementary Material

Supplemental Digital Content

## Supplementary Material

Supplemental Digital Content

## Supplementary Material

Supplemental Digital Content
