# Supplementary material for: Association between C-reactive protein level and subsequent risk of ovarian cancer: A meta-analysis of 13 cohorts in 1,852 ovarian cancer patients
Source: Medicine (Baltimore). 2020 Jan 31;99(5):e18821. doi: 10.1097/MD.0000000000018821 (PMC7004735; doi:10.1097/MD.0000000000018821)
Supplement: Supplemental Digital Content [file medi-99-e18821-s001.docx]

Table S1. Sensitivity analysis for all invasive ovarian cancer (highest versus lowest)

| Excluding study | RR and 95% CI | P-value | Heterogeneity (%) | P-value for heterogeneity |
| --- | --- | --- | --- | --- |
| NHS/NHS II | 1.37 (0.99-1.90) | 0.055 | 65.7 | 0.012 |
| WHS | 1.31 (0.97-1.76) | 0.081 | 60.1 | 0.028 |
| EPIC | 1.51 (1.22-1.88) | <0.001 | 0.0 | 0.566 |
| FMC | 1.33 (0.98-1.81) | 0.071 | 63.0 | 0.019 |
| Lundin 2009 | 1.43 (1.03-1.99) | 0.032 | 65.6 | 0.013 |
| PLCO | 1.29 (0.97-1.71) | 0.081 | 58.4 | 0.035 |
| McSorley 2007 | 1.30 (0.96-1.76) | 0.084 | 59.1 | 0.032 |
