# Supplementary material for: Association between C-reactive protein level and subsequent risk of ovarian cancer: A meta-analysis of 13 cohorts in 1,852 ovarian cancer patients
Source: Medicine (Baltimore). 2020 Jan 31;99(5):e18821. doi: 10.1097/MD.0000000000018821 (PMC7004735; doi:10.1097/MD.0000000000018821)
Supplement: Supplemental Digital Content [file medi-99-e18821-s002.docx]

Table S2. Sensitivity analysis for all invasive ovarian cancer (moderate versus lowest)

| Excluding study | RR and 95% CI | P-value | Heterogeneity (%) | P-value for heterogeneity |
| --- | --- | --- | --- | --- |
| NHS/NHS II | 1.19 (0.96-1.47) | 0.105 | 44.5 | 0.082 |
| NHS/NHS II | 1.19 (0.96-1.47) | 0.117 | 44.7 | 0.081 |
| WHS | 1.07 (0.91-1.26) | 0.387 | 12.0 | 0.337 |
| WHS | 1.11 (0.92-1.34) | 0.268 | 27.7 | 0.208 |
| EPIC | 1.26 (1.05-1.50) | 0.011 | 0.0 | 0.463 |
| FMC | 1.16 (0.94-1.42) | 0.162 | 42.1 | 0.098 |
| Lundin 2009 | 1.22 (0.99-1.50) | 0.067 | 40.6 | 0.108 |
| PLCO | 1.17 (0.95-1.43) | 0.143 | 43.5 | 0.088 |
| McSorley 2007 | 1.17 (0.94-1.44) | 0.154 | 43.1 | 0.091 |
