# Supplementary material for: Association between C-reactive protein level and subsequent risk of ovarian cancer: A meta-analysis of 13 cohorts in 1,852 ovarian cancer patients
Source: Medicine (Baltimore). 2020 Jan 31;99(5):e18821. doi: 10.1097/MD.0000000000018821 (PMC7004735; doi:10.1097/MD.0000000000018821)
Supplement: Supplemental Digital Content [file medi-99-e18821-s003.docx]

Table S3. Sensitivity analysis for serous ovarian cancer (highest versus lowest)

| Excluding study | RR and 95% CI | P-value | Heterogeneity (%) | P-value for heterogeneity |
| --- | --- | --- | --- | --- |
| NHS/NHS II | 1.51 (0.78-2.90) | 0.220 | 77.9 | 0.003 |
| WHS | 1.32 (0.74-2.35) | 0.346 | 72.7 | 0.012 |
| EPIC | 1.69 (1.11-2.58) | 0.014 | 25.6 | 0.258 |
| Lundin 2009 | 1.48 (0.75-2.92) | 0.252 | 77.1 | 0.004 |
| PLCO | 1.17 (0.76-1.82) | 0.478 | 59.5 | 0.060 |
