# Supplementary material for: Association between C-reactive protein level and subsequent risk of ovarian cancer: A meta-analysis of 13 cohorts in 1,852 ovarian cancer patients
Source: Medicine (Baltimore). 2020 Jan 31;99(5):e18821. doi: 10.1097/MD.0000000000018821 (PMC7004735; doi:10.1097/MD.0000000000018821)
Supplement: Supplemental Digital Content [file medi-99-e18821-s004.docx]

Table S4. Sensitivity analysis for serous ovarian cancer (moderate versus lowest)

| Excluding study | RR and 95% CI | P-value | Heterogeneity (%) | P-value for heterogeneity |
| --- | --- | --- | --- | --- |
| NHS/NHS II | 1.31 (0.90-1.90) | 0.154 | 59.4 | 0.031 |
| NHS/NHS II | 1.33 (0.91-1.94) | 0.137 | 59.8 | 0.029 |
| WHS | 1.18 (0.87-1.61) | 0.289 | 44.4 | 0.109 |
| WHS | 1.13 (0.86-1.49) | 0.381 | 30.9 | 0.203 |
| EPIC | 1.44 (1.08-1.93) | 0.014 | 14.0 | 0.325 |
| Lundin 2009 | 1.37 (0.94-2.00) | 0.099 | 58.9 | 0.032 |
| PLCO | 1.27 (0.90-1.78) | 0.174 | 57.9 | 0.037 |
